# Supplementary material for: Smartphone Distraction: Italian Validation of the Smartphone Distraction Scale (SDS)
Source: Int J Environ Res Public Health. 2023 Aug 2;20(15):6509. doi: 10.3390/ijerph20156509 (PMC10418316; doi:10.3390/ijerph20156509)
Supplement: Supplementary file 1 [file ijerph-20-06509-s001.zip › ijerph-2428233-supplementary.pdf]

International Journal of
Environmental Research and Public Health

MDPI

Supplementary Materials

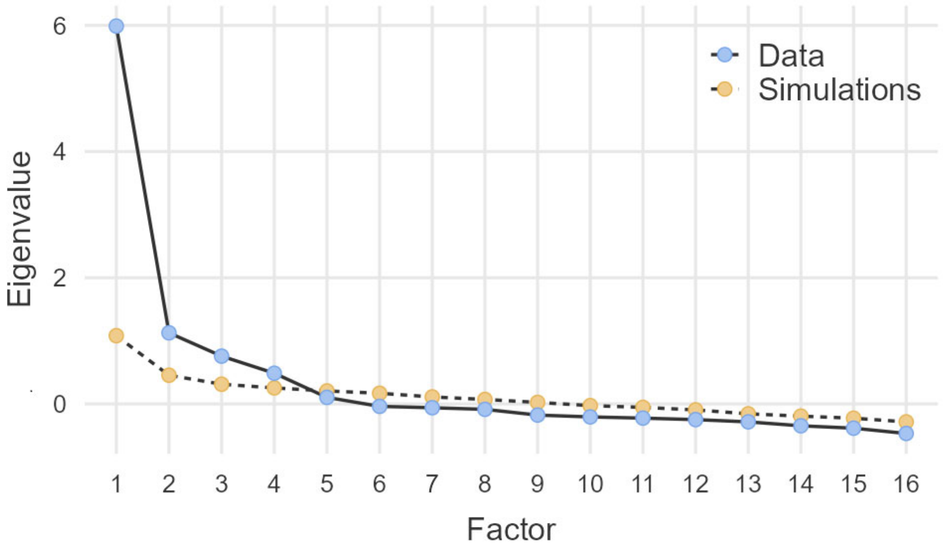

Figure S1. Scree plot and parallel analysis.

Table S1. Parameter estimates CFA – Model 1 – One factor.

Indicator	Estimate	SE	z value	p-value	95% CI lower	95% CI higher	R^2
Item 1	0.930	0.049	19.009	< .001	0.834	1.026	0.653
Item 2	0.973	0.053	18.455	< .001	0.869	1.076	0.628
Item 3	0.916	0.054	16.904	< .001	0.810	1.023	0.570
Item 4	0.946	0.052	18.211	< .001	0.845	1.048	0.617
Item 5	0.541	0.059	9.201	< .001	0.426	0.657	0.305
Item 6	0.686	0.058	11.911	< .001	0.573	0.799	0.448
Item 7	0.323	0.051	6.327	< .001	0.223	0.424	0.215
Item 8	0.184	0.045	4.135	< .001	0.097	0.272	0.096
Item 9	0.485	0.072	6.761	< .001	0.344	0.626	0.191
Item 10	0.274	0.075	3.655	< .001	0.127	0.421	0.055
Item 11	0.543	0.071	7.679	< .001	0.404	0.681	0.206
Item 12	0.385	0.063	6.088	< .001	0.261	0.509	0.157
Item 13	0.608	0.071	8.622	< .001	0.470	0.746	0.274
Item 14	0.647	0.068	9.540	< .001	0.514	0.779	0.287
Item 15	0.798	0.060	13.239	< .001	0.680	0.916	0.445
Item 16	0.755	0.065	11.542	< .001	0.626	0.883	0.360

Table S2. CFA – Model 2 - Parameter estimates.

factor	Indicator	Symbol	Estimate	SE	z value	p-value	95% CI lower	95% CI higher	R^2
Factor 1	Item 1	λ11	0.955	0.048	19.820	< .001	0.861	1.050	0.689
	Item 2	λ12	1.020	0.052	19.455	< .001	0.917	1.123	0.690
	Item 3	λ13	0.981	0.054	18.260	< .001	0.875	1.086	0.653
	Item 4	λ14	0.983	0.054	18.225	< .001	0.878	1.089	0.666
Factor 2	Item 5	λ21	0.653	0.064	10.201	< .001	0.528	0.779	0.445

	Item 6	λ22	0.884	0.056	15.783	< .001	0.774	0.993	0.744
	Item 7	λ23	0.392	0.054	7.320	< .001	0.287	0.497	0.316
	Item 8	λ24	0.258	0.045	5.776	< .001	0.170	0.345	0.188
Factor 3	Item 9	λ31	0.514	0.083	6.221	< .001	0.352	0.675	0.214
	Item 10	λ32	0.562	0.083	6.761	< .001	0.399	0.725	0.229
	Item 11	λ33	0.836	0.071	11.816	< .001	0.698	0.975	0.489
	Item 12	λ34	0.663	0.063	10.562	< .001	0.540	0.786	0.463
Factor 4	Item 13	λ41	0.823	0.068	12.137	< .001	0.690	0.955	0.502
	Item 14	λ42	0.910	0.063	14.415	< .001	0.786	1.034	0.570
	Item 15	λ43	0.974	0.056	17.491	< .001	0.865	1.084	0.663
	Item 16	λ44	0.958	0.060	16.079	< .001	0.841	1.074	0.580

Table S3. CFA -Model 3 – Parameter estimates**.**

factor	Indicator	Symbol	Estimate	SE	z value	p-value	95% CI lower	95% CI higher	R^2
First order									
Factor 1	Item 1	λ11	0.434	0.070	6.175	< .001	0.296	0.572	0.689
	Item 2	λ12	0.464	0.076	6.094	< .001	0.314	0.613	0.691
	Item 3	λ13	0.446	0.073	6.094	< .001	0.302	0.589	0.654
	Item 4	λ14	0.446	0.075	5.956	< .001	0.299	0.592	0.664
Factor 2	Item 5	λ21	0.395	0.059	6.729	< .001	0.280	0.510	0.447
	Item 6	λ22	0.531	0.065	8.230	< .001	0.405	0.658	0.738
	Item 7	λ23	0.237	0.042	5.684	< .001	0.156	0.319	0.318
	Item 8	λ24	0.156	0.030	5.242	< .001	0.098	0.215	0.190
Factor 3	Item 9	λ31	0.396	0.068	5.848	< .001	0.263	0.528	0.216
	Item 10	λ32	0.427	0.076	5.613	< .001	0.278	0.575	0.225
	Item 11	λ33	0.648	0.072	9.051	< .001	0.507	0.788	0.500
	Item 12	λ34	0.503	0.059	8.556	< .001	0.387	0.618	0.454
Factor 4	Item 13	λ41	0.583	0.056	10.407	< .001	0.473	0.692	0.504
	Item 14	λ42	0.643	0.059	10.953	< .001	0.528	0.758	0.569
	Item 15	λ43	0.688	0.054	12.672	< .001	0.581	0.794	0.661
	Item 16	λ44	0.677	0.057	11.880	< .001	0.565	0.789	0.580
Second order									
	Factor 1	γ11	1.962	0.400	4.910	< .001	1.179	2.745	0.794
	Factor 2	γ12	1.321	0.219	6.040	< .001	0.892	1.749	0.636
	Factor 3	γ13	0.839	0.151	5.564	< .001	0.543	1.134	0.413
	Factor 4	γ14	1.001	0.131	7.645	< .001	0.745	1.258	0.501
